# Editorial: Advances in combined modality treatments for prostate cancer

**DOI:** 10.3389/fsurg.2026.1918076

**Published:** 2026-09-17

**Authors:** Feifei Sun

**Affiliations:** Department of Pathology, Peking University People’s Hospital, Beijing, China

**Keywords:** androgen deprivation therapy, androgen receptor pathway inhibitor, chemotherapy, combined modality treatment, prostate cancer

Prostate cancer encompasses a broad clinical spectrum, from localized tumors that may be cured with local therapy to metastatic disease and lethal castration-resistant prostate cancer (CRPC). Its management has progressively shifted from reliance on a single modality toward coordinated combinations of surgery, radiotherapy, androgen deprivation therapy (ADT), androgen receptor pathway inhibitors (ARPIs), chemotherapy, and emerging biologically directed agents. Although treatment intensification has improved disease control, it has also made clinical decisions more complex. The essential question is therefore not simply whether therapies should be combined, but which combination is appropriate for a particular patient, disease state, and treatment goal.

In this Research Topic, “Advances in Combined Modality Treatments for Prostate Cancer,” we present a Research Topic of seven articles spanning the clinical course of prostate cancer. Together, they address three connected areas: optimization of local and neoadjuvant treatment, selection of intensified systemic therapy for metastatic hormone-sensitive prostate cancer (mHSPC), and investigation of resistance mechanisms and new therapeutic candidates in CRPC.

The first group of articles focuses on localized, high-risk, and locally advanced disease. Abu-Hijlih et al. compare radical prostatectomy with definitive radiotherapy plus ADT in a real-world cohort from a tertiary cancer center in Jordan. Both approaches achieved excellent and similar five-year cancer-specific and overall survival, while radiotherapy plus ADT provided better biochemical control. Although retrospective comparisons are influenced by patient selection and differing definitions of biochemical recurrence, this study adds valuable evidence from the Middle East and North Africa and supports individualized multidisciplinary treatment selection rather than a universal preference for one local modality.

Neoadjuvant treatment offers another approach to improving outcomes before definitive local therapy. In a systematic review and network meta-analysis of older adults with high-risk or locally advanced prostate cancer, Yuan et al. report that novel neoadjuvant hormonal therapy ranked highest for pathological outcomes, including surgical margin status and prostate-specific antigen (PSA) response, but was also associated with the greatest toxicity burden. This efficacy-toxicity trade-off is particularly relevant in older patients, for whom comorbidity, functional reserve, life expectancy, and treatment goals should complement chronological age in decision-making. Because improved pathological responses have not yet established a survival benefit, neoadjuvant intensification before prostatectomy remains investigational.

The second group addresses systemic treatment of mHSPC, where ADT alone has largely been replaced by doublet or triplet regimens. Zapatero et al. critically evaluate triplet therapy combining ADT, docetaxel, and a second-generation ARPI. Available evidence supports triplet treatment particularly for fit patients with high-volume and/or synchronous metastatic disease. However, direct randomized comparisons between triplet therapy and ADT plus an ARPI are lacking. Better predictive markers are therefore needed to distinguish patients who require chemotherapy-based intensification from those who may safely avoid docetaxel.

Treatment selection must also account for demands beyond conventional efficacy and toxicity endpoints. Using United States real-world data, Sentana-Lledo et al. show that adding docetaxel to an ARPI-based regimen was associated with more days spent managing prostate cancer and higher healthcare costs. Their findings highlight “time toxicity,” encompassing treatment visits, testing, travel, waiting, hospitalization, and management of adverse events. Such burdens do not negate the potential survival benefit of triplet therapy in selected patients, but they should form part of shared decision-making together with quality of life, financial consequences, and caregiver impact.

Real-world evidence is also essential for assessing whether trial results translate across populations and healthcare settings. Peng et al. report rapid and deep early PSA responses with rezvilutamide plus ADT in Chinese patients with high-volume mHSPC, with consistent activity across clinical subgroups and no new severe safety signal. These results support the feasibility of this regimen in routine Chinese practice. Longer follow-up is needed to determine how early PSA responses relate to radiographic progression, survival, and patient-reported outcomes.

The final group examines advanced disease and treatment resistance. Wang et al. review neuroendocrine differentiation as a mechanism of lineage plasticity and resistance under therapeutic pressure. They describe the interacting roles of genetic and epigenetic alterations, signaling pathways, metabolic reprogramming, non-coding RNAs, and the tumor microenvironment, as well as potential biomarkers and interventions. The heterogeneity and dynamic evolution of neuroendocrine prostate cancer emphasize the need to integrate pathology, imaging, circulating biomarkers, and genomic profiling to recognize phenotypic transformation and guide treatment adaptation.

Xing et al. explore resibufogenin as a candidate treatment for CRPC through compound screening, cellular experiments, network pharmacology, molecular docking, and molecular dynamics simulations. Resibufogenin inhibited prostate cancer cell proliferation and colony formation, increased reactive oxygen species, and altered apoptosis-related proteins, while BRAF and MAPK signaling emerged as possible mechanistic components. These preclinical findings provide a basis for further target validation, *in vivo* efficacy studies, and pharmacological and toxicological evaluation.

Collectively, these articles show that combined modality treatment should not mean indiscriminate therapeutic escalation. Its purpose is to integrate treatments according to disease stage, tumor biology, expected benefit, toxicity, patient fitness, treatment burden, access, and individual priorities. Future studies should directly compare competing combinations, validate predictive and resistance biomarkers, include diverse real-world populations, and incorporate patient-reported outcomes, geriatric assessment, financial toxicity, and time toxicity alongside survival.

Progress will depend on close collaboration among urologists, radiation and medical oncologists, pathologists, radiologists, geriatric specialists, translational scientists, and allied healthcare professionals. This collection provides timely evidence and perspectives toward more precise, effective, and sustainable combined modality treatment for prostate cancer.

